# Australasian Bronchiolitis Guideline: 2025 Update

**DOI:** 10.1111/jpc.70144

**Published:** 2025-07-20

**Authors:** Meredith L. Borland, Kate Loveys, Franz E. Babl, Elizabeth Cotterell, Libby Haskell, Sharon O’Brien, Ed Oakley, Catherine L. Wilson, Jane Alsweiler, David Armstrong, Simon S. Craig, Nigel W. Crawford, Dianne Crellin, Sonja Crone, Trevor Duke, Shane George, Christine Jeffries‐Stokes, Nidhi Krishnan, Anna Lithgow, Ken Peacock, Tomas Ratoni, Peter Richmond, Annie Smith, Rebecca Starkie, David Thomas, Alexandra Wallace, Michael Zhang, Emma Tavender, Stuart R. Dalziel, the Paediatric Research in Emergency Departments International Collaborative (PREDICT) Network, Australasia

**Affiliations:** ^1^ Emergency Department Perth Children’s Hospital Perth Western Australia Australia; ^2^ Divisions of Paediatrics and Emergency Medicine School of Medicine, University of Western Australia Perth Western Australia Australia; ^3^ Department of Paediatrics: Child and Youth Health School of Medicine, University of Auckland Auckland New Zealand; ^4^ Departments of Paediatrics and Critical Care University of Melbourne Parkville Victoria Australia; ^5^ Emergency Department Royal Children’s Hospital Parkville Victoria Australia; ^6^ Emergency Research, Clinical Sciences, Murdoch Children’s Research Institute Parkville Victoria Australia; ^7^ Armidale Rural Referral Hospital Armidale New South Wales Australia; ^8^ School of Rural Medicine and Tablelands Clinical School, University of New England Armidale New South Wales Australia; ^9^ Children’s Emergency Department Starship Children’s Hospital Auckland New Zealand; ^10^ Division of Emergency Medicine, Anaesthesia and Pain Medicine, Medical School University of Western Australia Perth Western Australia Australia; ^11^ Institute for Paediatric Perioperative Excellence, The University of Western Australia Perth Western Australia Australia; ^12^ Clinical Sciences, Murdoch Children’s Research Institute Parkville Victoria Australia; ^13^ Department of Respiratory and Sleep Medicine Monash Children’s Hospital Clayton Victoria Australia; ^14^ Department of Paediatrics Monash University Clayton Victoria Australia; ^15^ Paediatric Emergency Department Monash Medical Centre, Monash Health Clayton Victoria Australia; ^16^ Royal Children’s Hospital Melbourne Victoria Australia; ^17^ Murdoch Children’s Research Institute Melbourne Victoria Australia; ^18^ Department of Paediatrics University of Melbourne Melbourne Victoria Australia; ^19^ Clinical Sciences, Murdoch Children’s Research Institute Melbourne Victoria Australia; ^20^ University of Melbourne Melbourne Victoria Australia; ^21^ Department of Paediatrics Rotorua Hospital, Te Whatu Ora Lakes Rotorua New Zealand; ^22^ Gold Coast University Hospital Gold Coast Queensland Australia; ^23^ Rural Clinical School of Western Australia Kalgoorlie Western Australia Australia; ^24^ Kalgoorlie Regional Hospital, West Australian Country Health Service Kalgoorlie Western Australia Australia; ^25^ Queensland Children’s Hospital South Brisbane Queensland Australia; ^26^ Department of Paediatrics The Royal Darwin Hospital Darwin Northern Territory Australia; ^27^ Department of General Medicine Children’s Hospital at Westmead, Sydney Children’s Hospitals Network Westmead New South Wales Australia; ^28^ Northern NSW Local Health District New South Wales Australia; ^29^ Perth Children’s Hospital Perth Western Australia Australia; ^30^ University of Western Australia Perth Western Australia Australia; ^31^ Southland Hospital Invercargill New Zealand; ^32^ Department of General Practice and Primary Care Melbourne Medical School, Faculty of Medicine, Dentistry and Health Sciences, University of Melbourne Melbourne Victoria Australia; ^33^ Department of General Medicine Women’s and Children’s Hospital Adelaide South Australia Australia; ^34^ Department of Paediatrics Waikato Hospital, Te Whatu Ora Waikato Health NZ Hamilton New Zealand; ^35^ Emergency Department John Hunter Hospital New Lambton Heights New South Wales Australia; ^36^ Australian Catholic University (ACU) Sydney New South Wales Australia; ^37^ Department of Surgery School of Medicine, The University of Auckland Auckland New Zealand

**Keywords:** bronchiolitis, guideline, management, paediatric, respiratory

## Abstract

**Aim:**

To provide updated evidence‐based clinical guidance in the management of infants with bronchiolitis presenting to emergency departments (EDs), general paediatric, or intensive care units (ICUs) in Australia and Aotearoa New Zealand (AoNZ) following the first publication in 2016.

**Method:**

The Paediatric Research in Emergency Departments International Collaborative (PREDICT) network guideline working group appraised, summarised, and updated evidence from 1 January 2000 to 24 January 2024 addressing 41 questions (30 from the 2016 guideline and 11 new questions for 2025). Recommendations were developed using GRADE methodology and revised after a period of external consultation.

**Results:**

The literature search identified 26 467 citations with 431 included in 41 recommendations providing 11 new and 7 key updates. The key changes included: (i) refinement of the clinical features of bronchiolitis, (ii) addition of new risk factors for severity of illness, (iii) advice on the role of biomarkers for unexpected deterioration or admission to ICU, (iv) guidance on glucocorticoids in SARS‐CoV‐2 co‐infection, (v) guidance on combined glucocorticoids/inhaled epinephrine in severe bronchiolitis requiring ICU level care, (vi) refinement of oxygen saturation targets, (vii) guidance on humidified high flow therapy and continuous positive airway pressure, (viii) recommendation on use of RSV prevention therapies/immunisations for babies and mothers.

**Conclusion:**

The updated Australasian Bronchiolitis Guideline provides clinicians across Australasian settings with the latest evidence‐based guidance on the management of the commonest condition in infancy requiring hospital admission.

## 1 Introduction

Bronchiolitis is one of the most common reasons for hospital admission in Australian and Aotearoa New Zealand (AoNZ) infants [1, 2]. It is an acute respiratory condition generally occurring seasonally in winter months, most often associated with a Respiratory Syncytial Virus (RSV) infection [3] and typically affects infants < 12 months of age [4]. The condition begins with signs of an upper respiratory tract infection (e.g., rhinorrhoea, nasal congestion, cough), followed by signs of a lower respiratory tract infection, including respiratory distress and the presence of diffuse crackles and/or wheeze. Bronchiolitis may also involve hypoxaemia, apnoea, increased respiratory and heart rate, the use of accessory muscles, feeding difficulties, irritability and/or lethargy [4].

To inform the management of infants presenting to hospital or hospitalised with bronchiolitis in Australia and AoNZ, the Paediatric Research in Emergency Departments International Collaborative (PREDICT) formed a Guideline Development Committee to develop the first Australasian Bronchiolitis Guideline published online in 2016 [5, 6]. The guideline was prompted by the lack of Australasian‐specific, evidence‐based guidance on the management of bronchiolitis in infants, with data indicating variation in clinical practice in this acute setting [7, 8]. It provided 31 recommendations covering 22 investigations and therapies.

In 2022, PREDICT commenced a planned update of the Australasian bronchiolitis guideline, to incorporate evidence since the first guideline. The scope was expanded to include new topics on preventative therapies for RSV, treatment of SARS‐CoV‐2 co‐infection, as well as broadening the recommendations to high dependency (HDU)/intensive care (ICU) level care.

### 1.1 Aims and Objectives

The 2025 guideline update aims to provide evidence‐based guidance for a target audience of clinicians managing infants (aged < 12 months) with bronchiolitis, who have presented to an emergency department (ED), admitted to a general paediatric ward or an ICU/HDU (requiring treatment up to the point of intubation and mechanical ventilation) in the Australasian setting. This article reports on the update of the original 2016 guideline [6].

## 2 Materials and Methods

The Australasian Bronchiolitis Guideline was prospectively registered on the Guideline International Network (GIN) library [9], and PROSPERO (CRD42023463917) [10]. The Appraisal of Guidelines Research and Evaluation (AGREE) and Check‐Up reporting checklists were followed. Extensive details of the methodology, including lessons learned for guideline developers and reporting checklists, are available in a companion article [11]. The full guideline report and evidence profiles are freely available on the PREDICT website [12, 13].

The PREDICT Bronchiolitis Guideline Advisory Group (GAG), consisting of five medical (MLB, SRD, FEB, EC, EO) and two nursing specialists (LH, SO) in general paediatrics and paediatric emergency medicine (PEM), plus three methodology experts (KL, EJT, CW), from Australia and AoNZ, was established to have overall responsibility for determining the updated guideline strategy and process, overseeing the evidence review, and managing the development of recommendations through to publication. In addition, 19 clinical and academic experts in paediatric emergency medicine, immunology, neonatology, intensive care, general paediatrics, and primary care were invited to form the Guideline Development Committee (GDC) whose role was to determine the scope, provide feedback during the evidence review, develop the recommendations, and vote to finalise the recommendations.

### 2.1 Scope and Process

Key clinical questions in population, intervention, comparator, outcome (PICO) format relevant to the scope, target audience, aims and objectives were developed. In total, 41 PICO questions were incorporated into the 2025 guideline, including 30 from the 2016 guideline and 11 new questions. Systematic searches were developed with and performed by a subject librarian (Royal Children's Hospital, Melbourne, VIC, Australia), using electronic databases Ovid MEDLINE, Ovid EMBASE, PubMed, CINAHL, and the Cochrane Library (last search 24 January 2024) (Supporting Information). The results were limited to English and by publication date from 2000 onwards. The search strategy from the initial guideline was adapted to include terms on the new PICO questions. Supplementary searches were performed for the new questions, backdated to the search period of the initial guideline (2010–2014). The included articles of the 2016 guideline were screened for eligible ICU evidence.

The quality of the body of evidence was assessed using Grading of Recommendations Assessment, Development and Evaluation (GRADE) methodology with GRADEpro GDT software (McMaster University and Evidence Prime Inc., Hamilton, ON, Canada) [14, 15, 16]. Recommendations were developed using the GRADE evidence‐to‐recommendation framework [16]. The strength of recommendations varied from strong, conditional, weak, or consensus‐based (in the absence of evidence). Recommendations considered the evidence quality, the balance of associated benefits and harms, the resource implications, feasibility in the Australasian context, acceptability, values and preferences, and equity and human rights, with the recommendation strength representing the degree to which the GDC were confident that the desirable effects of the action outweighed the undesirable effects. Evidence profiles, including evidence‐to‐recommendation tables per recommendation, are available elsewhere [13].

Finalisation of the recommendation wording was undertaken through consensus discussion and voting by the GAG/GDC at three guideline development meetings held on 1st December 2023 (virtual), 23rd February 2024 (in‐person), and 17th May 2024 (virtual), where 80% agreement was required. Consensus was reached on all topics.

Interest‐holder consultation was performed over 8 weeks to gain feedback on the recommendations and ensure the acceptability, feasibility, and applicability of the recommendations to Australasian consumers of the guideline (clinicians, policymakers, patients and families). This process is described in detail in our companion methodology paper [11]. Ten colleges, five societies, and 13 hospitals and local governance groups in Australia and AoNZ were invited to provide consultation. Formal endorsement was sought from the two specialist colleges credentialing care in Australia and AoNZ hospitals (Australasian College for Emergency Medicine and Royal Australasian College of Physicians). All organisations approached focused on paediatrics, emergency medicine, intensive care, and primary care in Australia and AoNZ. This process was supplemented by in‐depth interviews of families with a recent hospital experience of bronchiolitis in metropolitan and rural hospitals in Australasia to understand values and preferences for bronchiolitis care, and inform the recommendations [11].

## 3 Results

**FIGURE 1:**
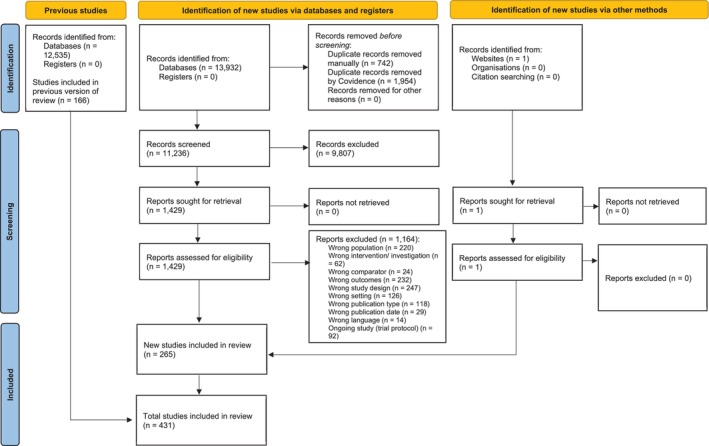
PRISMA flow chart. *Source:* Page MJ, et al. BMJ 2021;372:n71. doi: https://doi.org/10.1136/bmj.n71. This work is licensed under CC BY 4.0. To view a copy of this license, visit https://creativecommons.org/licenses/by/4.0/ .

The updated literature search was conducted on two occasions (19 and 21 June 2023, and 24 January 2024) and identified 13 932 new manuscripts, in addition to the 12 535 manuscripts from 2016, giving a total of 26 467 manuscripts screened, of which 431 studies (265 new, 166 from 2016) were included (Figure 1). Evidence tables were prepared for the 25 questions that informed evidence summaries and recommendations. Nine recommendations were made relating to diagnosis and 32 relating to management, with nine strong, 22 conditional, 15 weak, and eight consensus‐based recommendations (Table 1). The full guideline is available at https://www.predict.org.au/bronchiolitis‐guideline/ with the additional Bedside Guideline aid for busy clinicians developed [12].

**TABLE 1:** Recommendations of the 2025 Australasian Bronchiolitis Guideline.

Topic	Recommendation	Recommendation strength
Diagnosis		
Physical examination and history (Q1)	1. Consider a diagnosis of bronchiolitis in an infant if they have an upper respiratory tract infection (rhinorrhoea/nasal congestion, and/or cough), followed by the onset of a lower respiratory tract infection with one or more of respiratory distress (tachypnoea and/or retractions), or presence of diffuse crackles and/or wheeze, with or without the presence of fever. Additional signs and symptoms can include feeding difficulties, vomiting, dehydration, hypoxaemia, lethargy, uncommonly (< 5%) diarrhoea, and rarely (< 2%) apnoea.	1. Weak 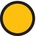
Risk factors (Q2)	2. Clinicians should take into account the following risk factors for more serious illness when assessing and managing infants with bronchiolitis: Gestational age < 37 weeks;* Younger chronological age at presentation;* Prenatal and/or postnatal exposure to tobacco smoke;* Reduced breastfeeding exposure;* Faltering growth/slow weight gain (failure to thrive); Comorbidities including congenital heart disease, chronic lung disease, chronic neurological condition, congenital diaphragmatic hernia, trisomy 21, and other genetic disorders; Being an Indigenous infant^**†**^; Being an economically disadvantaged infant; Timing and severity of illness onset at hospital presentation. *Clinicians should judge these as risk factors on a continuous scale; with higher risk of poor outcomes associated with lower gestational age, lower chronological age, fewer days of breastfeeding exposure, and greater tobacco smoke exposure. ^**†**^Indigenous status, in itself, is unlikely to confer risk but there remains a correlation in Australia and Aotearoa New Zealand with ethnicity and severe bronchiolitis outcomes, independent of socioeconomic status, potentially reflecting the ongoing impacts of colonisation, remote geographic isolation, and the institutional racism in our health systems.	2. Strong 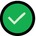
CXR (Q3a–c)	3a. Do not routinely use CXR in infants presenting or admitted to hospital with bronchiolitis. 3b. Consider CXR in infants with an unexpected deterioration* and/or a clinical course not consistent with bronchiolitis, including concerns regarding the presence of sepsis, pneumonic consolidation, pneumothorax, empyema, immunodeficiency, pleural effusion, or significant cardiac abnormalities. *Unexpected deterioration refers to an unexpected requirement for an escalation of care. 3c. Consider CXR in infants with bronchiolitis in high dependency/intensive care settings, where there is clinician diagnostic concern regarding possible sepsis, pneumonic consolidation, pneumothorax, empyema, immunodeficiency, pleural effusion or significant complication of other diseases (e.g., heart failure with congenital heart disease), in order to guide treatment options.	3a. Conditional 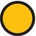 3b. Consensus‐based 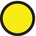 3c. Consensus‐based 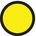
Laboratory tests (Q4a–c)	4a. (i) Do not routinely use laboratory tests for infants presenting to hospital or hospitalised with bronchiolitis, including bacteriological testing of urine or blood. (ii) Consider glucose and/or sodium levels during assessment in infants with bronchiolitis and poor feeding, evidence of dehydration or altered mental state. 4b. Consider use of biomarkers (e.g., FBC, CRP, PCT), urine testing, and blood cultures for the diagnosis of serious bacterial co‐infection for infants with unexpected deterioration during hospitalisation with bronchiolitis. 4c. Consider use of biomarkers (e.g., FBC, CRP, PCT) and blood cultures for diagnosis of serious bacterial co‐infection for infants being admitted to ICU with bronchiolitis.	4a. (i) Conditional 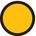 (ii) Consensus‐based 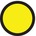 4b. Consensus‐based 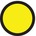 4c. Weak 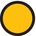
Virological investigations (Q5)	5. Do not routinely use viral testing in infants presenting to hospital or hospitalised with bronchiolitis, including testing undertaken solely for cohorting of patients.	5. Conditional 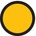
Management		
Bronchiolitis scoring systems (Q6)	6. Do not routinely use a formal bronchiolitis severity scoring system to predict need for admission or hospital length of stay in infants presenting or admitted to hospital with bronchiolitis.	6. Weak 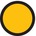
Criteria for safe discharge (Q7)	7. Safe discharge from hospital (either from the ED or ward) for infants with bronchiolitis should take into account risk factors (R2), the distance of the family's residence from the hospital and their ability to return, parental health literacy, and the timing of the hospital presentation relative to the natural history of bronchiolitis (R1). Consider patients suitable for safe discharge from hospital when the following criteria are met: 1. Infant is clinically stable (defined as with mild to moderate stable respiratory effort). 2. For an infant who has not received oxygen/respiratory support and/or with SpO_2_ ≥ 95%, there is no need to continue to observe for maintenance of oxygen saturations and may be considered for discharge based on criteria below. For an infant who has received oxygen/respiratory support and/or with SpO_2_ ≤ 94%, they should be observed for maintenance of oxygen saturations in air at the following levels for 3–4 h, including a period of sleep: i. for infants aged ≥ 6 weeks with no underlying health conditions, for maintenance of SpO_2_ ≥ 90%; ii. for infants aged < 6 weeks, or infants aged < 12 months with an underlying health condition, for maintenance of SpO_2_ ≥ 92%. 3. All Infants irrespective of presentation to ED or on inpatient ward should be maintaining adequate oral intake of fluids and feeds of at least 1/2 of usual volume with adequate output (> 1/2 of usual wet nappies). 4. Parents and/or caregivers should feel confident to manage the infant with bronchiolitis at home. 5. Parents and/or caregivers are educated and provided with written information on possible deterioration and when to return for healthcare review. 6. Social situation allows discharge to home. The following factors should be considered: social factors, the time of day and suitable transport availability. 7. Arrange local follow‐up where appropriate.	7. Weak 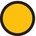
Beta2 agonists (Q8a–b)	8a. Do not use beta2 agonists in infants (< 12 months of age) presenting to hospital or hospitalised with bronchiolitis. 8b. Do not use beta2 agonists in infants (< 12 months of age) presenting to hospital or hospitalised with bronchiolitis, with a personal or family history of atopy outside of a RCT.	8a. Strong 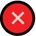 8b. Strong 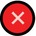
Adrenaline/epinephrine (Q9)	9. Do not use adrenaline/epinephrine in infants presenting to hospital or hospitalised with bronchiolitis.	9. Strong 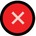
Hypertonic saline (Q10)	10. Do not routinely use nebulised hypertonic saline in infants presenting to hospital or hospitalised with bronchiolitis outside of an RCT.	10. Weak 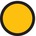
Glucocorticoids (Q11a–c)	11a. Do not use systemic or local glucocorticoids in infants with bronchiolitis*. *For guidance on the use of glucocorticoids when SARS‐CoV‐2 infection is present, refer to R22b ‘SARS‐CoV‐2 treatment.’ 11b. Do not use glucocorticoids for the routine treatment of infants with bronchiolitis with a positive response to beta2 agonists or other markers of a latter asthmatic phenotype outside of a RCT. Beta2 agonists should not be used in infants aged < 12 months (see Q8a,b). 11c. (i) Do not routinely use a combination of systemic or local corticosteroids and adrenaline/epinephrine in infants presenting to or hospitalised with moderate bronchiolitis outside of the ICU setting. (ii) Consider using a combination of systemic or local corticosteroids and adrenaline/epinephrine in infants with severe bronchiolitis requiring ICU level care.	11a. Strong 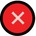 11b. Strong 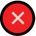 11c. (i) Conditional 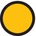 (ii) Conditional 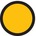
Supplemental oxygen and saturation targets (Q12a–b)	12a. Consider the use of supplemental oxygen in the treatment of hypoxaemic* infants with bronchiolitis. *For definitions of hypoxaemic and target oxygen saturation levels, see Q12b (‘Oxygen saturation targets’). 12b. Consider the use of supplemental oxygen in infants with bronchiolitis if their oxygen saturation is: Persistently < 90%, for infants aged ≥ 6 weeks; Persistently < 92%, for infants aged < 6 weeks, or infants aged < 12 months with an underlying health condition.	12a. Conditional 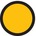 12b. Weak 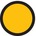
Continuous pulse oximetry (Q13)	13. Do not routinely use continuous pulse oximetry for medical management of non‐hypoxaemic infants (SpO_2_ ≥ 90% for infants ≥ 6 weeks age, or SpO_2_ ≥ 92% for infants < 6 weeks age, or infants aged < 12 months an underlying health condition), with bronchiolitis not receiving oxygen, or stable infants receiving low‐flow oxygen, who are not at risk of apnoea.	13. Conditional 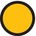
HF therapy (Q14)	14. (i) Do not routinely use HF therapy in infants with mild or moderate bronchiolitis who are not hypoxaemic.* (ii) Do not routinely use HF therapy as a first‐line therapy in infants with moderate bronchiolitis who are hypoxaemic.* (iii) Consider HF therapy in infants with bronchiolitis who are hypoxaemic, * and who have failed low flow oxygen. (iv) Consider HF therapy in infants with bronchiolitis with severe disease prior to CPAP. * For otherwise healthy infants aged ≥ 6 weeks: SpO_2_ persistently < 90%. For infants aged < 6 weeks, or infants aged < 12 months with an underlying health condition: SpO_2_ persistently < 92%.	14. (i) Conditional 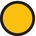 (ii) Conditional 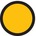 (iii) Conditional 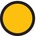 (iv) Conditional 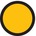
Chest physiotherapy (Q15)	15. Do not routinely use chest physiotherapy in infants with bronchiolitis.	15. Conditional 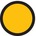
Suctioning (Q16a–b)	16a (i). Do not routinely use nasal suction in the management of infants with bronchiolitis. (ii). Consider using superficial suctioning in infants who have respiratory distress or feeding difficulties due to upper airway secretions. 16b. Do not routinely use deep nasal suctioning for the management of infants with bronchiolitis.	16a (i). Conditional 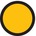 (ii). Conditional 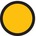 16b. Weak 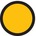
Nasal saline (Q17)	17 (i). Do not routinely use nasal saline drops in the management of infants with bronchiolitis. (ii). Consider a trial of intermittent nasal saline drops at time of feeding in infants with reduced feeding.	17 (i). Conditional 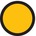 (ii). Conditional 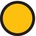
CPAP (Q18)	18. Consider using CPAP therapy in infants with bronchiolitis and impending or severe respiratory failure, and/or with severe illness.	18. Conditional 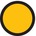
Antibiotic medication (Q19a–c)	19a. Do not routinely use antibiotics for the treatment of infants with bronchiolitis. 19b. Do not routinely use azithromycin for treatment of bronchiolitis in infants admitted to hospital. 19c. Do not routinely use antibiotics for the treatment of bronchiolitis in infants who are at risk of developing bronchiectasis (due to known risk factors such as virus type (e.g., Adenovirus), Indigenous ethnicity, socioeconomic disadvantage).	19a. Conditional 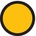 19b. Weak 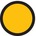 19c. Weak 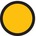
Non‐oral hydration (Q20a–e)	20a. Use supplemental hydration for infants with bronchiolitis who cannot maintain hydration orally. 20b. (i) Use either NG or IV routes for non‐oral hydration in infants admitted to hospital with bronchiolitis requiring supplemental hydration. (ii) Consider NG as the preferred first method of non‐oral hydration in infants with moderate bronchiolitis requiring supplemental hydration. (iii) Consider either continuous or bolus methods of NG non‐oral hydration with oral rehydration solution, breast milk, or formula in infants admitted to hospital with bronchiolitis requiring an NG. 20c. Consider fluid restriction at 50%–75% of recommended maintenance due to the risk of fluid overload from syndrome of inappropriate antidiuretic hormone secretion, and hyponatremia in bronchiolitis. Monitor for signs of overhydration. 20d. Consider using either 0.9% sodium chloride (normal saline) with 5% glucose, or balanced fluid (e.g., Plasma‐lyte 148 or Hartmann's solution) with 5% glucose, for use as maintenance fluid in infants admitted to hospital with bronchiolitis requiring IV hydration. For younger infants aged up to 4 weeks corrected with bronchiolitis, consider 10% glucose, or monitoring of blood sugar levels if receiving 5% glucose. 20e. (i) Consider enteral feeding (NG or oral), if tolerated, in infants receiving high flow. (ii) Consider continuous NG feeding in infants receiving CPAP who are not judged at imminent risk of intubation.	20a. Strong 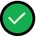 20b. (i) Strong 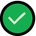 (ii) Weak 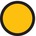 (iii) Conditional 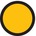 20c. Consensus‐based 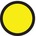 20d. Consensus‐based 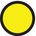 20e. (i) Weak 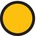 (ii) Consensus‐based 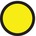
Infection control practices (Q21)	21. (i) Use hand hygiene practices for the management of infants with bronchiolitis. (ii) Consider multicomponent infection control practices for the management of infants with bronchiolitis. (iii) Consider cohorting of infants admitted to inpatient wards with bronchiolitis.	21. (i) Strong 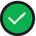 (ii) Weak 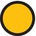 (iii) Weak 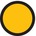
SARS CoV‐2 co‐infection and treatment (Q22a–b)	22a. Do not routinely use SARS‐CoV‐2 status to stratify increased risk for deterioration in infants with bronchiolitis. SARS CoV‐2 infection or co‐infection does not appear to place infants at increased risk of severe outcome from bronchiolitis. 22b. (i) Consider use of dexamethasone in hypoxic patients presenting with bronchiolitis who are also positive for SARS‐CoV‐2 co‐infection. (ii) Consider use of remdesivir in immunosuppressed infants who are also positive for SARS‐CoV‐2 infection.	22a. Weak 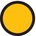 22b. (i) Consensus‐based 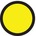 (ii) Consensus‐based 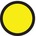
Prevention		
Infant RSV monoclonal antibody prophylaxis (Q23)	23. (i) Consider use of monoclonal antibodies (palivizumab or nirsevimab) during RSV season in infants at increased risk of severe complications with bronchiolitis; chronic lung disease, congenital heart disease, and infants born very preterm (< 32 wGA). (ii) Consider universal nirsevimab as a population‐based approach to reduce morbidity due to RSV bronchiolitis.	23. (i) Conditional 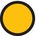 (ii) Conditional 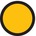
Maternal active RSV immunisation (Q24)	24. Consider universal maternal antenatal immunisation with a RSV prefusion F protein‐based vaccine as a population‐based approach to reduce morbidity due to RSV bronchiolitis.	24. Conditional 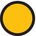
Infant active RSV immunisation (Q25)	25. Do not routinely use universal infant RSV immunisation.	25. Weak 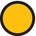

*Note:* Strong against: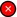; Conditional/weak (for or against):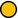; Strong for: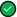; Consensus‐based: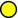.

Abbreviations: CPAP, continuous positive airway pressure; CRP, C‐reactive protein; CXR, chest x‐ray; ED, emergency department; FBC, full blood count; GP, general practise; HDU, high dependency unit; HF, high flow; ICU, intensive care unit; IM, intramuscular; IV, intravenous; MDI, metered dose inhaler; NG, nasogastric; PCT, procalcitonin; RCT, randomised controlled trial; RSV, respiratory syncytial virus; SARS‐CoV‐2, severe acute respiratory syndrome coronavirus 2; UTI, urinary tract infection, wGA, weeks, gestational age.

### 3.1 Key Changes Between the 2016 and 2025 Guidance

Seven key changes were made to recommendations from 2016 relating to physical examination, risk factors for more serious illness, laboratory testing, criteria for safe discharge, glucocorticoids, supplemental oxygen and saturation targets, and infection control practises, along with 11 recommendations for new topics in 2025. Table 2 summarises the changed and new recommendations between the initial and updated guideline.

**TABLE 2:** A summary of key changes in the recommendations between the initial and updated guideline.

Topic	No.	Change	Summary of changes	2016 Recommendation
Physical exam and history	1	✓	The key clinical signs and symptoms of bronchiolitis have not changed. However, additional clinical signs and symptoms have been added to the recommendation: feeding difficulties, vomiting, dehydration, hypoxaemia, lethargy, uncommonly (< 5%) diarrhoea, and rarely (< 2%) apnoea.	Infants can be diagnosed with bronchiolitis if they have an upper respiratory tract infection followed by onset of respiratory distress with fever, and one or more of: cough, tachypnoea, retractions and diffuse crackles or wheeze on auscultation. (NHMRC: C, GRADE: Weak)
Risk factors	2	✓	Additional risk factors have been added to the recommendation, including the presence of trisomy 21, economic disadvantage, CDH, other genetic disorders, and the timing of illness onset at hospital presentation. In the 2025 update, clinicians are encouraged to view gestational age, chronological age, breastfeeding and tobacco smoke exposure (pre and postnatal) as continuous risk factors (where risk of serious illness is increased with lower gestational or chronological age, less breastfeeding exposure, and more tobacco smoke exposure).	Clinicians should consider as risk factors for more serious illness: gestational age < 37 weeks; chronological age at presentation < 10 weeks; exposure to cigarette smoke; breastfeeding for < 2 months; failure to thrive; having chronic lung disease; having chronic heart and/or chronic neurological conditions; being Indigenous ethnicity, and should take these into account when managing infants with bronchiolitis. (NHMRC: C, GRADE: Conditional)
CXR	3b	NA	New topic to the 2025 guideline update.	NA
	3c	NA	New topic to the 2025 guideline update.	NA
Laboratory tests	4a	✓	The recommendation to perform urine testing for suspected urinary tract infection in infants with bronchiolitis and a fever was removed to reflect the updated evidence. However, urine tests may be considered to inform diagnoses of serious bacterial co‐infection in infants with unexpected deterioration (see R4b). The recommendation was updated to report that glucose and/or sodium levels may be considered during assessment in infants with bronchiolitis and poor feeding, evidence of dehydration or altered mental state.	There is no role for blood tests in managing infants presenting to hospital and hospitalised with bronchiolitis. Routine bacteriological testing of blood and urine is not recommended. In infants < 2 months of age presenting to hospital or hospitalised with bronchiolitis with a temperature > 38°, there is a low risk of UTI. If clinical uncertainty exists, clinicians may consider collecting a urine sample for microscopy, culture, and sensitivity looking for the concurrent presence of UTI. (NHMRC: D, GRADE: Conditional)
	4b	NA	New topic to the 2025 guideline update.	NA
	4c	NA	New topic to the 2025 guideline update.	NA
Criteria for safe discharge	7	✓	In the 2025 update, a prescriptive discharge criteria and flow chart was developed. The criteria for safe discharge were revised to include specific oxygen saturation targets and indicators of adequate feeding, and the criteria were tailored to ED and ward discharge. Additional detail on the social factors surrounding discharge, such as parent/caregiver education on bronchiolitis and confidence to manage bronchiolitis from home, transport, and arrangement of local follow‐up (if needed) were added.	Oxygen saturations, adequacy of feeding, age (infants < 8 weeks), and lack of social support should be considered at the time of discharge as a risk for representation. There is insufficient evidence to recommend absolute discharge criteria for infants attending the ED, or hospitalised with bronchiolitis (NHMRC: Practice Point, GRADE: Weak)
Glucocorticoids	11c	✓	The 2025 update states that combined glucocorticoid and adrenaline/epinephrine therapy may be considered in infants with severe bronchiolitis who are requiring ICU level care. The 2025 guidance is otherwise consistent with the 2016 guideline in advising against the routine use of combined therapy in infants with moderate bronchiolitis outside of the ICU setting.	Do not administer a combination of systemic or local glucocorticoids and adrenaline/epinephrine to infants presenting to hospital or hospitalised with bronchiolitis. (NHMRC: D, GRADE: Weak)
Saturation targets	12b	✓	In the 2025 update, it is recommended to use supplemental oxygen in infants with bronchiolitis if SpO_2_ is persistently < 90% in infants aged ≥ 6 weeks. For infants < 6 weeks of age, or < 12 months of age with an underlying health condition, supplemental oxygen should be used if SpO_2_ is persistently < 92%.	In uncomplicated bronchiolitis oxygen supplementation should be commenced if the oxygen saturation level is sustained at a level < 92%. At oxygen saturation levels of 92% or greater, oxygen therapy should be discontinued. (NHMRC: C, GRADE: Conditional)
Non‐oral hydration	20b	✓	In the updated recommendation, further detail was provided on the types of NG hydration that may be given. Clinicians can consider either continuous or bolus methods of NG non‐oral hydration with oral rehydration solution, breast milk, or formula in infants admitted to hospital with bronchiolitis requiring an NG. NG is the preferred first method of non‐oral hydration in infants with moderate bronchiolitis requiring supplemental hydration.	Both NG and IV routes are acceptable means for non‐oral hydration in infants admitted to hospital with bronchiolitis. (NHMRC: B, GRADE: Strong).
	20c	✓	The recommendation has been updated to provide more specific guidance on fluid restriction. Clinicians can consider fluid restriction at 50%–75% of recommended maintenance due to the risk of fluid overload from syndrome of inappropriate antidiuretic hormone secretion (SiADH), and hyponatremia in bronchiolitis. Clinicians are also encouraged to monitor for signs of overhydration.	There is insufficient evidence to recommend a specific proportion of maintenance fluid. There is a risk of fluid overload therefore judicious and vigilant use of hydration fluid is required and regular review is recommended (NHMRC: Practice point, GRADE: Weak).
	20d	NA	New topic to the 2025 guideline update.	NA
	20e	NA	New topic to the 2025 guideline update.	NA
Infection control practises	21	✓	In addition to hand hygiene practises and cohorting of patients in wards, the 2025 update recommends that multicomponent infection control measures may be considered whilst managing infants with bronchiolitis (e.g., use of gowns, masks).	Hand hygiene is the most effective intervention to reduce hospital acquired infections and is recommended. There is inadequate evidence for benefits in cohorting infants with bronchiolitis. (NHMRC: D, GRADE: Weak)
SARS‐CoV‐2 co‐infection	22a	NA	New topic to the 2025 guideline update.	NA
SARS‐CoV‐2 treatment	22b	NA	New topic to the 2025 guideline update.	NA
Monoclonal antibody therapy	23	NA	New topic to the 2025 guideline update.	NA
Maternal RSV immunisation	24	NA	New topic to the 2025 guideline update.	NA
Infant RSV immunisation	25	NA	New topic to the 2025 guideline update.	NA

*Note:* The recommendations were not reported as changed in instances where there were minor changes to the wording of the recommendation, but the recommended action had not changed.

Of note, the 2025 guideline recommendations with key changes from 2016 include:

#### 3.1.1 Physical Examination and History

Key additional clinical signs and symptoms were added to the recommendation of assessment of severity, which include feeding difficulties, vomiting, dehydration, hypoxaemia, lethargy, uncommonly (< 5%) diarrhoea, and rarely (< 2%) apnoea.

#### 3.1.2 Risk Factors for More Severe Disease

There is no role for the routine use of a formal bronchiolitis severity scoring system to predict the need for hospital admission or length of stay in infants presenting or admitted to hospital with bronchiolitis. New additional risk factors in 2025 include the presence of trisomy 21, economic disadvantage, pre‐natal exposure to cigarette smoke, congenital diaphragmatic hernia, unspecified genetic disorders, and the timing of the hospital presentation in relation to the duration of illness symptoms. Clinicians should judge these as continuous risk factors with the greater the risk of poorer outcomes from bronchiolitis.

Indigenous status, in itself, is unlikely to confer risk but there remains a correlation in Australia and AoNZ between ethnicity and severe bronchiolitis outcomes, independent of socioeconomic status, potentially reflecting the ongoing impacts of colonisation, remote geographical isolation, and the institutional racism in our health systems.

#### 3.1.3 Laboratory (Blood, Urine) Testing

In assessing an infant < 12 months of age with bronchiolitis and fever, the revised recommendation has removed the component of the 2016 guideline advising consideration of performing a urine test for suspected urinary tract infection (UTI), reflecting updated evidence [17]. The 2025 recommendation reiterates that there is no role for routine blood tests in managing infants presenting to hospital and/or hospitalised with bronchiolitis. Glucose and/or sodium levels may be considered during assessment in infants with bronchiolitis with poor feeding, evidence of dehydration, or altered mental state. Routine bacteriological testing of blood and urine is not recommended.

With the expanded scope of the guideline to infants with unexpected deterioration and/or admission to ICU, there is a recommendation to consider obtaining blood tests (e.g., Full Blood Count (FBC), C‐Reactive Protein (CRP), Procalcitonin (PCT) and blood cultures) for the diagnosis of serious bacterial co‐infection for infants with unexpected deterioration during hospitalisation or being admitted to ICU with bronchiolitis.

#### 3.1.4 Criteria for Safe Discharge

**FIGURE 2:**
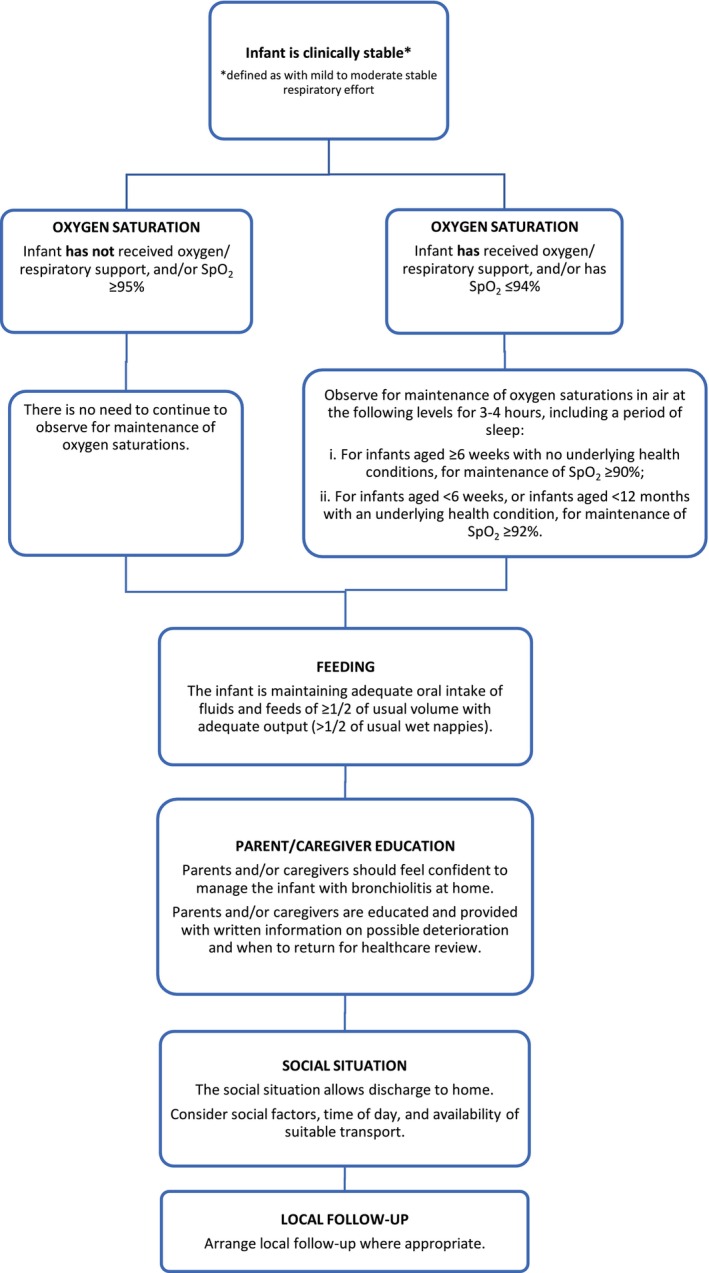
Criteria for safe discharge. SpO_2_, Peripheral oxygen saturation.

Recommendations relating to safe discharge from the ED and inpatient services were revised to include specific oxygen saturation targets and indicators of adequate feeding, with criteria tailored to reflect differences between ED and inpatient ward discharge (Figure 2). Additional detail on the social factors surrounding discharge, including the need to provide parent/caregiver education on bronchiolitis and take into consideration factors including parental/caregiver health literacy, their confidence in managing bronchiolitis at home, access to appropriate transport, and capacity for local follow‐up has been incorporated.

#### 3.1.5 Glucocorticoids

Recommendation against the use of systemic glucocorticoids for infants presenting to hospital or hospitalised with bronchiolitis remains in the revised guideline. However, recommendations have been made for consideration of single therapy systemic glucocorticoids in those infants with SARS‐CoV‐2 co‐infection, as well as consideration of combination therapy of glucocorticoids with inhaled epinephrine/adrenaline in “infants with severe bronchiolitis requiring ICU level care” based on recent evidence [18, 19].

#### 3.1.6 Oxygen Saturation Targets

Consider use of supplemental oxygen in the treatment of hypoxaemic infants with bronchiolitis. Supplementary oxygen should not be used for work of breathing alone. Do not routinely use continuous pulse oximetry for medical management of non‐hypoxaemic infants with bronchiolitis who are not receiving supplemental oxygen, or in stable infants receiving low flow oxygen therapy. Continuous pulse oximetry should be considered with the presence of apnoeas, or risk factors for apnoea, in this disease setting.

The recommendation for the commencement of supplemental oxygen therapy has been updated for hypoxaemic infants with bronchiolitis [20, 21].

- In infants ≥ 6 weeks, it is recommended to use supplemental oxygen in infants with bronchiolitis if SpO_2_ is persistently < 90%.
- In infants < 6 weeks, or < 12 months with an underlying health condition, supplemental oxygen should be used if SpO_2_ is persistently < 92%.

#### 3.1.7 Humidified High Flow Therapy

**FIGURE 3:**
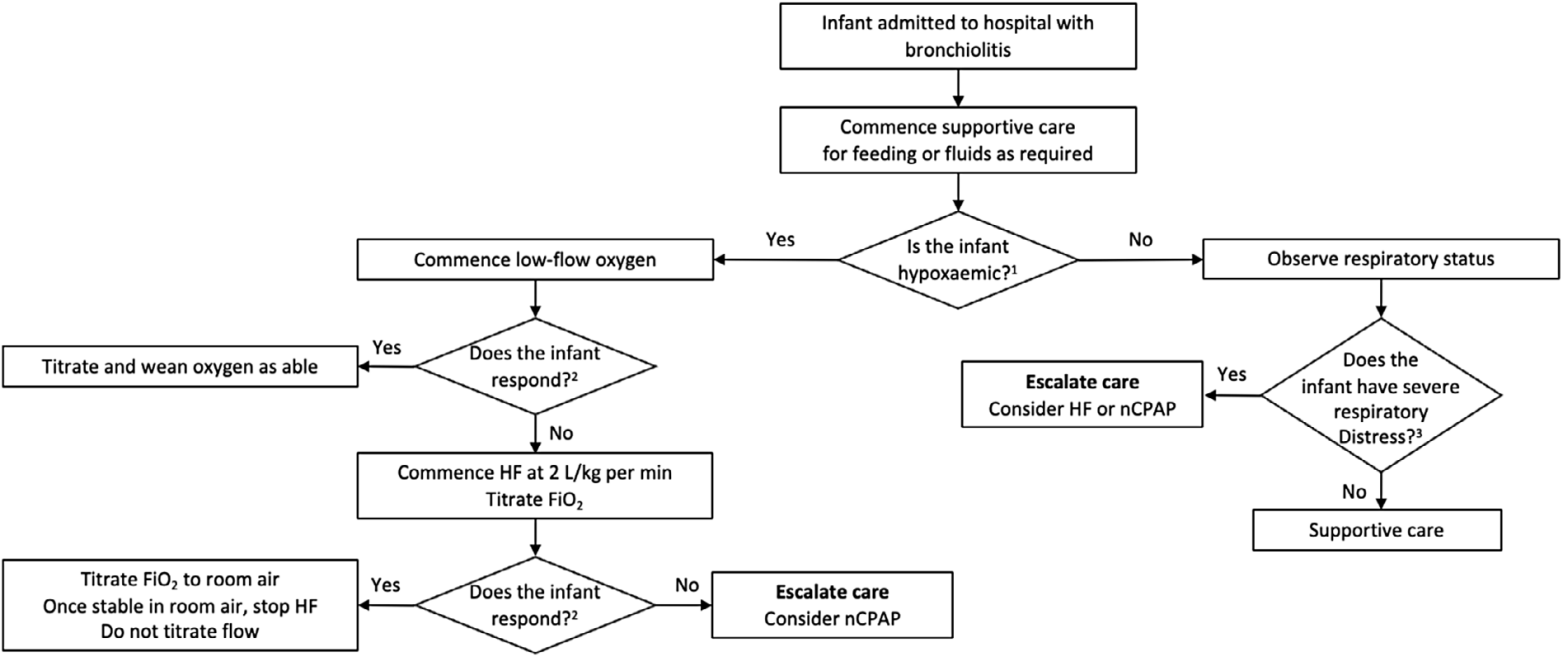
An evidence‐based approach to the use of respiratory support in infants with bronchiolitis. FiO_2_, fractional concentration of inspired oxygen; HF, humidified high flow; nCPAP, nasal continuous positive airway pressure. ^1^For otherwise healthy infants aged ≥ 6 weeks: SpO_2_ persistently < 90%. For infants aged < 6 weeks, or infants < 12 months with an underlying health condition: SpO_2_ persistently < 92%. ^2^Response to therapy (low‐flow or HF oxygen therapy) is determined by a reduction in respiratory rate, a reduction in heart rate, or a paediatric early warning score within 4–5 h of commencing therapy. ^3^If at any time, the infant has severe respiratory distress, escalate care. Respiratory distress is a subjective finding. Severe respiratory distress is a level where a senior clinician determines that escalation in care is required, transferring the patient to the emergency department resuscitation area, paediatric ward resuscitation area, high dependency unit, or intensive care unit. Junior staff should escalate concerns regarding severe respiratory distress to senior colleagues.

Whilst there are no changes in the recommendations for the use of humidified high flow (HF) therapy in 2025, there has been a substantial maturing of the evidence, and we emphasise not to routinely use HF therapy in non‐hypoxaemic infants with mild or moderate bronchiolitis. In addition, we do not recommend the routine use of HF therapy as the first‐line oxygen therapy in infants with moderate bronchiolitis who are hypoxaemic but to consider HF use when infants have failed low flow oxygen therapy (Figure 3).

#### 3.1.8 Continuous Positive Airway Pressure (CPAP)

A new conditional recommendation for the use of CPAP in infants with bronchiolitis has been formulated to include those with impending respiratory failure, and/or with severe disease.

#### 3.1.9 Hydration

Hydration status may be considered inadequate if there is < 50% of normal intake or evidenced by 5% weight loss or hypernatremia (if tested). After treatment of hypoxaemia, feeding is often improved. Supplemental hydration should be provided to infants with bronchiolitis who cannot maintain hydration orally.

The recommendation relating to the use of fluid supplements has been refined to include:

- The NG route should be the preferred first method in infants with moderate bronchiolitis requiring supplemental hydration with consideration of either continuous or bolus methods of NG hydration using oral rehydration solution/breast milk or formula.
- Consider fluid restriction (between 50% and 66%), due to the risk of fluid overload. Careful monitoring of signs of over‐hydration (facial and eye‐lid oedema, weight increase) and under‐hydration is needed.
- Whilst enteral feeding (oral or NG) is still the first option, clinicians may consider using either 0.9% sodium chloride or balanced intravenous fluid with 5% glucose for non‐enteral hydration in infants with bronchiolitis who require intravenous hydration. For younger infants aged 4 to 8 weeks, consider monitoring blood sugar levels or increasing to 10% glucose.
- Infants receiving HF therapy (or those receiving CPAP therapy who are not judged to be at imminent risk of intubation) may receive enteral feed via the oral/NG route (if tolerated), utilising standard fluid restriction (between 50% and 75%) in the management of bronchiolitis.

#### 3.1.10 Infection Control

The 2025 recommendation continues to recommend the use of hand hygiene practises, with the addition of recommendations to cohort patients with bronchiolitis and to consider multicomponent infection control measures such as gowns and/or masks.

#### 3.1.11 Discharge Planning and Community‐Based Management

For infants with bronchiolitis, safe discharge from the hospital (ED or ward) should consider risk factors for severe illness, the distance of the family's residence from the hospital, their ability to return, their health literacy, and the timing of the hospital presentation relative to the natural history of bronchiolitis.

Infants should be considered as safe for discharge from hospital when the criteria are met from Figure 2. These criteria incorporate clinical stability, oxygen saturation and support requirements, feeding difficulties, parent/caregiver ability to manage the illness from home and education on deterioration, the social situation of the family, and arrangement of local follow‐up if needed.

Parents and caregivers should be educated about the illness, the expected progression, and when and where to seek further medical care if needed. Written Bronchiolitis Discharge Information (hard copy and/or electronic) should be provided to parents and caregivers.

#### 3.1.12 SARS‐CoV‐2 Co‐Infection and Treatment

The 2025 guideline recommends that for situations where virological testing has been performed and the infant with bronchiolitis is found to be positive for SARS‐CoV‐2 infection, SARS‐CoV‐2 infection or co‐infection does not appear to place infants at increased risk of severe outcome from bronchiolitis. In subgroups of infants with bronchiolitis associated with SARS‐CoV‐2 infection:

- Consider the use of dexamethasone in hypoxaemic patients presenting with bronchiolitis who are also positive for SARS‐CoV‐2 co‐infection.
- Consider the use of remdesivir in immunosuppressed infants who are also positive for SARS‐CoV‐2 infection.

Note that the guideline does not make a recommendation to perform viral testing for the purpose of assessing for a SARS‐CoV‐2 infection in infants with bronchiolitis. Rather, this guidance is to inform the hospital management of infants in situations where SARS‐CoV‐2 infection has been detected from viral testing that may have been performed for other reasons (e.g., local hospital requirements).

#### 3.1.13 RSV Prevention

During the RSV season: (a) Consider the use of monoclonal antibodies (palivizumab or nirsevimab) in infants at increased risk of severe complications with bronchiolitis including those with; (i) chronic lung disease; (ii) congenital heart disease; or (iii) infants born very pre‐term (< 32 weeks gestational age), (b) Consider universal nirsevimab as a population‐based approach to reduce morbidity due to RSV bronchiolitis, c. Consider universal maternal antenatal immunisation with an RSV prefusion F protein‐based vaccine as a population‐based approach to reduce morbidity due to RSV bronchiolitis, d. Do not routinely use universal infant RSV immunisation. To date, there are no approved active vaccines for RSV in infants. Routine interventions in Australia and AoNZ should be approved by the Therapeutic Goods Administration and Medsafe prior to consideration in a universal immunisation programme.

Within Australia and AoNZ, most infants with bronchiolitis requiring hospital care are seen in metropolitan, regional and rural centres. In these hospitals, infants with severe disease may need to be managed for some time in an ED or inpatient paediatric ward, prior to transfer to a tertiary children's hospital ICU or managed in an adult ICU without transfer. The appropriate setting for delivery of care should reflect resources and skills that are available at a given centre, rather than a specific physical location or label. For mild disease, where no hydration or respiratory support is required, the infants are usually managed in ED and/or in primary care. For moderate disease where hydration support and/or oxygen therapy (low flow or HF oxygen) are required, care can be safely delivered in a ward environment. An infant with severe bronchiolitis requires a higher nursing ratio (e.g., 1:1 or 1:2). This generally requires transport to HDU/ICU care or a higher‐level facility with these resources.

## 4 Discussion

The 2025 PREDICT Australasian Bronchiolitis GDC has reviewed and revised the inaugural 2016 Australasian Bronchiolitis Guideline to ensure that recommendations and practises align with an expanding and extensive literature base. We have made 41 recommendations for the management of infants with bronchiolitis in Australia and AoNZ supported by the current evidence base. All our recommendations are developed using GRADE methodology after an extensive literature search. Despite this, there remain many areas where there are conditional, weak, or consensus‐based recommendations supported by low‐quality evidence at best.

Areas in the previous guideline which left some uncertainty for clinicians related to the initiation of oxygen therapy based on oxygen saturation level and guidance on the use of HF and CPAP therapy for supportive care. Recent studies [21, 22] have reassured clinicians that a more lenient use of oxygen supplementation for infants with bronchiolitis is safe. The use of HF therapy at the time of the previous guideline was based on low‐quality evidence from observational studies mainly conducted in ICU settings. Subsequently [23, 24] large RCTs in Australasian EDs and inpatient wards have demonstrated that HF therapy is an appropriate therapy after failure of low‐flow oxygen therapy to maintain saturations above the recommended level [25, 26]. Our literature review identified two systematic reviews of HF therapy in bronchiolitis [27, 28], of apparent acceptable quality, undertaken at similar times, but with conflicting results (one supported early use of HF therapy, whilst one did not). These differences appeared driven by methodological decisions, including differences in included and excluded articles. On review, the GAG had additional concerns with both systematic reviews and an additional RCT had subsequently been published which was excluded from both. As the GAG did not have confidence in either of the published systematic reviews, we undertook an up‐to‐date independent systematic review and meta‐analysis of the primary articles in both systematic reviews to robustly inform our recommendations: that HF therapy should be used as rescue therapy in hypoxaemic infants who have failed low‐flow oxygen therapy.

A recent study on the combination therapy of glucocorticoids with inhaled epinephrine/adrenaline in the ICU setting has suggested synergy of these two medications, resulting in a reduction in severity of illness and the need for interventions for infants with severe bronchiolitis [18, 19]. Further studies are underway across Canada, Australia and AoNZ to determine if there may be a reduction in hospitalisation in infants with mild/moderate bronchiolitis who receive similar combination therapy in the ED setting.

Our 2025 recommendations are in keeping with an update to the UK NICE guideline in 2021, which outlines oxygen saturation targets and the provision of oxygen therapy consistent with our recommendations [20]. In addition, a recent literature review evaluating the evidence since the 2014 American Academy of Paediatrics (AAP) Bronchiolitis guideline also confirmed the themes of our update [29]. This review highlighted that there are many areas in development for the management of bronchiolitis, including the combined use of dexamethasone/inhaled epinephrine/adrenaline [19] and the use of hypertonic saline/albuterol (salbutamol) [30, 31].

### 4.1 Updating the Guideline

Following the release of the guideline, several priority topics will be adapted to a “living” format with more frequent evidence surveillance and refining of the recommendations as appropriate. Topics under consideration for this format are those in which there is upcoming, notable evidence that may require key adjustments to the recommendations. The guideline will then be updated in its entirety when appropriate.

### 4.2 Strengths and Limitations

The scope of the revised 2025 guideline was broadened to include patients requiring intensive care management up to (but not including) intubation and mechanical ventilation. We have also included preventative therapies for RSV, including immunotherapy and vaccinations as well as exploring the impact of SARS‐CoV‐2 co‐infection on the management of bronchiolitis. These changes have resulted in the subsequent recommendations being highly generalisable to all infants with bronchiolitis managed in primary care, EDs and throughout inpatient areas. The recommendations were developed through a rigorous methodological process consisting of systematic reviews, GRADE methodology, interest‐holder consultation and interviews, and the involvement of a diverse guideline development panel of 29 interest‐holders from varied healthcare settings in Australasia [11]. An in‐depth reflection on our methodology and lessons learned for national guideline developers and methodologists is included in our companion paper [11]. As part of the GRADE evidence‐to‐decision framework, the cost and resourcing associated with the proposed action informed the recommendation strength and were documented in evidence profiles [13]. Where appropriate, some topics further included cost‐effectiveness as a systematic review outcome. A monitoring and evaluation framework, including key clinical indicators and an audit tool, is being developed to measure hospital performance against the recommendations. These will be made freely available to all hospitals in Australia and AoNZ through the PREDICT website (https://www.predict.org.au/bronchiolitis‐guideline/).

A limitation of the guideline is that the scope was restricted to hospital management of bronchiolitis. As a result, it does not include recommendations for bronchiolitis management in primary care or telehealth, or guidance for long‐term follow‐up of infants after discharge. However, bronchiolitis remains the most common reason for infants to be admitted to hospital, and clear evidence management is required in this environment as the majority of healthcare costs associated with bronchiolitis occur in hospital. Regardless, the evidence present is likely highly relevant to primary care and telehealth. Local healthcare providers may be best placed to develop the most appropriate follow‐up protocols for their setting, due to variation in the availability of healthcare resources, practices, and population characteristics (e.g., cultural background). The guideline scope did not cover ipratropium bromide. Although it is commonly used in older infants with viral‐induced wheeze or asthma, there is evidence that it is not often used (2.2%; less than use of salbutamol, adrenaline, corticosteroids, and antibiotics) for infants aged < 12 months with bronchiolitis in Australasian settings [32]. However, it may be worthwhile considering the evidence for ipratropium bromide in a future guideline update for the purposes of preventing inappropriate use. Lastly, the guideline scope only covered infants aged under 12 months with bronchiolitis, whilst bronchiolitis occurs after this age, and the evidence presented is relevant to this age group; there is greater diagnostic uncertainty for bronchiolitis in infants aged 12–24 months, and the evidence should be interpreted with increased caution.

### 4.3 Conclusions

The Australasian Bronchiolitis Guideline has been updated to provide clinicians across Australasian settings with the latest evidence‐based guidance on the management of the commonest condition in infancy requiring hospital admission.

## Conflicts of Interest

D.A., D.C., E.C., N.C., S.C., T.D., S.C., L.H., C.J.S., N.K., A.L., K.L., K.P., T.R., A.S., R.S., D.T., E.T., A.W., C.W., M.Z. have no conflicts of interest to declare. M.L.B., S.R.D., F.E.B., S.O., and E.O.’s institutions have received equipment from Fisher and Paykel Healthcare to support bronchiolitis research. S.R.D. has received funding from Fisher and Paykel Healthcare for travel to an international meeting discussing high flow therapy. P.R.’s institution has received funding for (i) R.S.V. maternal and paediatric vaccination research, (ii) RSV monoclonal antibodies research in infants and high‐risk children, (iii) development of a live commensal bacteria for the prevention of otitis media and viral respiratory infections and (iv) Virtual Lectures to RSV investigators in Merck monoclonal antibody trial (Merck) and Canadian health care workers on RSV prevention (Astra Zeneca). S.G.’s institution has received funding from Fisher and Paykel Healthcare to support bronchiolitis research. J.A.’s institution has received funding for R.S.V. monoclonal antibodies research in infants and high‐risk children. None of the authors receive any personal financial benefits from industry sponsors.

## Supporting information


**Data S1.** Supporting Information.
